# Parvovirus B19 and Cellular Transcriptome Dynamics in Differentiating Erythroid Progenitor Cells

**DOI:** 10.3390/v18010039

**Published:** 2025-12-25

**Authors:** Erika Fasano, Niccolò Guglietta, Federica Bichicchi, Ilaria Gasperini, Elisabetta Manaresi, Giorgio Gallinella

**Affiliations:** 1 Department of Pharmacy and Biotechnology, University of Bologna, 40138 Bologna, Italy; erika.fasano2@unibo.it (E.F.); niccolo.guglietta2@unibo.it (N.G.); federica.bichicchi2@unibo.it (F.B.); ilaria.gasperini3@unibo.it (I.G.); elisabetta.manaresi@unibo.it (E.M.); 2 Microbiology Unit, IRCCS Azienda Ospedaliero-Universitaria di Bologna, 40138 Bologna, Italy

**Keywords:** Parvovirus B19, erythroid progenitor cells, cytofluorimetry, FISH, high-throughput sequencing, bioinformatics, transcriptome analysis, interaction networks

## Abstract

Parvovirus B19 (B19V) is a human ssDNA virus with ample pathogenic potential. It is characterized by a selective tropism for erythroid progenitor cells (EPC), exerting a cytotoxic effect with blockade of erythropoiesis. In our work, we investigated both viral and cellular expression profile in the course of infection of EPCs cultures via mRNA high throughput sequencing technology (HTS) and a dedicated bioinformatic pipeline, reconstructing both the viral and cellular transcriptome and their variations. A productive infection was confirmed as restricted to EPCs expressing mature differentiation markers and the specific receptor for virus VP1u region. mRNA HTS reconstructed the viral transcriptome in terms of localization and abundance of the different mRNA species, detailing the differential expression profile of B19V among early or late times in the course of infection. Analysis of cellular transcriptome indicated that variation was mainly driven by the cellular differentiation process, with the virus impacting to a lesser level, but still clearly separating infected vs. non-infected profiles. At early times post-infection, variations were typical of cellular sensing of viral infection and aimed at the induction of an antiviral state. At later times in the course of infection, the cellular population showed induction of an inflammatory response, related to TNF and IL-10, and a transition to adaptive immunity with evidence of upregulation of genes involved in MHC-II presentation. This dual-transcriptome analysis on infected EPCs population can lay the ground for future research aimed at a better definition of the pathogenetic mechanisms of B19V.

## 1. Introduction

In the *Parvoviridae* family, Parvovirus B19 (B19V) is a widely diffuse human virus with ample pathogenic potential [1]. Commonly associated with infectious erythema and acute/chronic arthralgia, B19V is typically responsible for pathologies of the hematopoietic compartment, such as erythroid aplastic crises and chronic erythrocyte aplasia. In addition, the virus is involved in several other systemic or organ diseases, including acute or chronic myocarditis, while the infection, if contracted by pregnant women, poses a high risk of intrauterine transmission and damage to the fetus. The pathogenesis is mainly a consequence of the selective tropism of B19V for progenitor cells in the erythroid lineage, in which B19V exerts a cytotoxic effect with a blockade of erythropoiesis, and of the triggering of inflammatory responses in non-erythroid tissues. A characteristic of B19V is its ability to establish long-term persistence in the host, creating a diffuse archived DNA reservoir in diverse tissues, whereas the possibility of viral reactivation to productive infection is still poorly defined [2].

**Figure 1:**
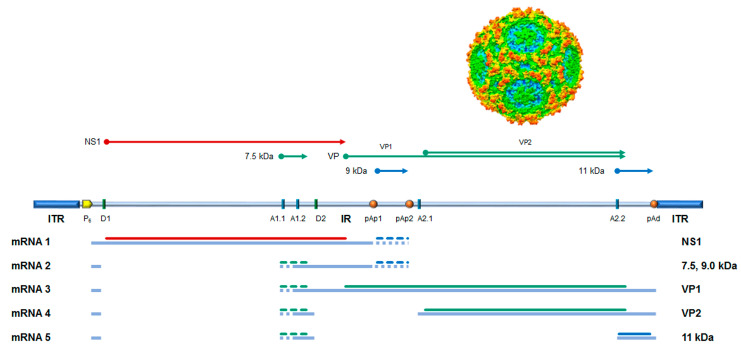
B19V genome organization, transcription map, encoded proteins, and capsid structure. Schematic diagram of B19V genome indicating the two inverted terminal regions (ITR) and the internal region (IR), with the distribution of cis-acting functional sites (P6, promoter; pAp1, pAp2, proximal cleavage-polyadenylation sites; pAd, distal cleavage-polyadenylation site; D1 and D2, splice donor sites; A1.1, A1.2, A2.1, and A2.2, splice acceptor sites). Bottom: Simplified transcription map of B19V genome, indicating the five classes of mRNAs (mRNA 1–5) with respective alternative splicing/cleavage forms (dashed) and their coding potential (colors indicate different reading frames). Top: Coding sequences for the viral proteins. NS1: non-structural protein NS1; VP: structural proteins, colinear VP1 and VP2, assembled in a T = 1 icosahedral capsid; and 7.5 kDa, 9.0 kDa, and 11 kDa: minor non-structural proteins. Adapted from [4]. Capsid structure from [5].

The genome is a linear single-stranded DNA molecule, 5596 nt long, organized in a unique internal region, containing all the open reading frames (ORFs), and flanked by inverted terminal regions that serve as origins of replication. In its internal region, the genome presents two major ORFs, on the left side coding for the non-structural protein NS1 and on the right side coding for the two colinear capsid proteins, VP1 and VP2. Minor ORFs can encode other non-structural proteins, namely the 11 kDa, 9.0, and 7.5 kDa proteins. The genome is encapsidated in an icosahedral shell composed of 60 protein subunits in a T = 1 arrangement, about 25 nm in diameter, composed of 5–10% VP1 and 90–95% VP2 proteins [3]. (Figure 1).

The B19V replicative cycle follows a complex series of events [6]. Upon virion attachment, penetration, and translocation into the nucleus, the viral genome is released, becoming accessible to the cellular replicative and transcriptional machinery. Conversion of the incoming single-stranded DNA into a double-stranded DNA forms a transcriptional active template, directed by a unique promoter at the left end of the genome. Transcription of the B19V genome leads to the production of five mRNA classes, with a total of twelve mature mRNAs species, depending on the alternative processing events of pre-mRNA [7]. Regulation of pre-mRNA processing is coupled to the onset of an active replication complex [8,9,10], with the resulting effect of a biphasic expression pattern [11,12]. In an early phase, mRNA processing is mostly confined to the proximal cleavage-polyadenylation sites and subsequent expression of the NS1 protein, thus leading to genome replication; in a late phase, following genome replication, mRNA processing extends to the distal cleavage-polyadenylation site, with subsequent expression of capsid proteins and assembly of mature virions. B19V proteome is limited, including six proteins at most [6,13]: the non-structural NS1 protein, main effector of viral replication and interaction with cell machinery, the two colinear capsid proteins VP1 and VP2, and the smaller non-structural proteins including the more characterized 11 kDa protein, essential for viral replication, and the elusive 9.0 and 7.5 kDa proteins.

The marked tropism for erythroid progenitor cells in bone marrow strictly depends on the differentiation stage and proliferation rate of this cell population. This restriction is the result of a combination of cell susceptibility, linked to the presence on the cell membrane of specific receptor moieties for B19V virions, and permissiveness, mainly linked to erythroid-specific intracellular signaling pathways. In other cell types that can be infected by B19V, notably endothelial or connective tissue cells, internalization may not depend on receptor interaction, expression of viral genome may be partial, and the replicative cycle is not normally productive. Thus, B19V-induced pathogenetic mechanisms differ between infection of erythroid progenitors, mostly leading to apoptosis, and non-erythroid tissues, mostly leading to activation of an inflammatory response [6].

In vitro, B19V can infect and be propagated in primary erythroid progenitor cells (EPCs) cultures, differentiated from peripheral blood mononuclear cells, which constitute a most appropriate model to study B19V replication and impact on cells [14,15]. EPCs cultures are a complex but well-characterized cell system, where the replicative cycle and expression profile of B19V have been described in detail [16]. EPC cultures also provide a suitable model to study the impact of viral infection on cellular expression profile. The progress of massive, high-throughput sequencing (HTS) and bioinformatic analytical tools offer novel opportunities to investigate virus–cell interaction in a comprehensive, systematic approach. In our present work, we sought to characterize both viral and cellular expression profile in the course of infection of EPC cultures. To this aim, we exploited high throughput sequencing technology and focused on reconstructing both the viral transcriptome in infected cells and the variations in the cell expression profile in the course of infection.

## 2. Materials and Methods

### 2.1. Virus

Infectious B19V was generated via nucleofection in UT7/EpoS1 cells of a cloned synthetic construct corresponding to a complete B19V genome (GenBank: KY940273.1) and amplification in successive rounds of infection in EPCs as described [17]. For infection experiments, a viral stock at a titer of 10^10^ genome copies (gec) per mL was used.

### 2.2. Cells

Erythroid progenitor cells (EPCs) were generated in vitro from peripheral blood mononuclear cells (PBMC) obtained from the leukocyte-enriched buffy coats of a healthy blood donor (Immunohematology and Transfusion Service, IRCSS S.Orsola, Bologna, authorization 0070755/1980/2014). Availability was granted under conditions complying with Italian privacy law; neither specific ethics committee approval nor written consent from donors was required for this research project. Isolated PBMC were cultured in IMDM supplemented with 20% serum substitute BIT 9500 and enriched with erythropoietic growth factors. The cells were maintained at 37 °C, 5% CO_2_, and used for infection experiments at day 8, as described [16]. For the reported experiments, cells were obtained from the same donor and a single donation unit and further processed in triplicate series as described.

### 2.3. Cytofluorimetric Analysis

In vitro cultured EPCs were characterized using flow cytometry (FACSCalibur, BD Biosciences, San Jose, CA, USA). Aliquots of 5 × 10^5^ EPCs were stained with antibodies specific for erythroid differentiation markers (CD36, CD71, GPA) and via VP1u binding for VP1u receptor. CD36 and CD71 expression was evaluated by phycoerythrin (PE)-labeled monoclonal antibodies (BD Biosciences). GPA expression was evaluated by monoclonal mouse anti-GPA (BD Biosciences) followed by anti-mouse-PE antibody (DakoCytomation, Glostrup, Denmark). VP1u receptor (VP1uR) expression was evaluated by binding of recombinant VP1u-FLAG (kindly provided by C. Ros, Bern, Switzerland) [18] followed by rabbit anti-FLAG antibody (Agilent Technologies, Cedar Creek, TX, USA); immunofluorescence detection was carried out with secondary goat anti-rabbit antibody-Dylight488 (Immunoreagents). Data were analyzed using the Cell Quest Pro Software v5.2.1 (BD Biosciences).

### 2.4. Infection and Sampling

For infection of EPCs, aliquots of 10^6^ cells in 100 µL of minimal medium were incubated in the presence of B19V to a multiplicity of infection (moi, expressed as geq/cell) of 10^3^ gec/cell for 2 h at 37 °C. After removal of inoculum virus, cells were incubated at 37 °C, 5% CO_2,_ in complete growth medium, at an initial density of 10^6^ cells/mL. In parallel, EPCs were cultured in the absence of the virus as reference. For both conditions, sampling was carried out at 2, 16 and 48 h post-infection (hpi) in triplicate series.

### 2.5. FISH Analysis

At the indicated time points post-infection, equal amounts of cell cultures, corresponding to 1.5 × 10^6^ cells, were collected and processed by flow-FISH assay for the detection of viral nucleic acids, as described [19]. Cells were fixed in PBS-paraformaldehyde 0.5% at 4 °C, permeabilized by resuspending in PBS containing 0.2% saponin, and finally resuspended in 50 μL of a hybridization solution containing 70% formamide. A digoxigenin-labeled DNA probe mixture specific for B19V nucleic acids was generated by the random priming method on the cloned full-length genomic DNA template (Dig-High Prime, Merck, Darmstadt, Germany). The probe mixture was separately denatured at 95 °C for 5 min, then added at 400 ng/mL to the cell suspension, previously heated at 70 °C for 5 min. Hybridization occurred at 37 °C for 12 h; then, cells were washed at RT for 15 min in 1x Stringent Wash (Zytovision, Bremerhaven, Germany). For the detection of the hybrids, the cell suspension was incubated with a FITC conjugate anti-digoxigenin antibody (Roche, Basel, Switzerland), washed twice in PBS, and resuspended in PBS for subsequent flow cytometry analysis.

### 2.6. qPCR and qRT-PCR

At the indicated time points post-infection, equal amounts of cell cultures, corresponding to 1.5 × 10^5^ cells, were collected. Pelleted cells were then processed by using the Maxwell Viral Total Nucleic Acid kit on a Maxwell MDx platform (Promega, Milan, Italy) to obtain a purified total nucleic acid fraction in elution volumes of 150 µL. A quantitative evaluation of target nucleic acids was carried out by qPCR and qRT-PCR assays in a Rotor-Q system (Qiagen, Milan, Italy). For the analysis of B19V DNA, aliquots of the eluted nucleic acids (corresponding to ~500 cells) were directly amplified in a qPCR assay (Maxima SYBR Green qPCR Master Mix, Thermo Fisher Scientific, Monza, Italy). For the analysis of B19V RNA, parallel aliquots were first treated with the Turbo DNAfree reagent (Thermo Fisher Scientific) before amplification in a qRT-PCR assay (QuantiNova SybrGreen RT-PCR Mix, Qiagen). Standard cycling programs were used, followed by a melting curve analysis to define the Tm of amplified products. Primer pairs (Table 1) were selected to allow normalization for cellular DNA (genomic 18S rDNA), quantitation of viral DNA, total viral RNA, and selected subsets of viral RNA. Quantitative evaluation of targets was obtained by absolute quantitation on external calibration curves, with a linear range of quantitation extending from 10^2^ to 10^8^ target copies, as described [11,12].

### 2.7. High-Throughput Sequencing

#### 2.7.1. DNA Sequencing

Collected samples were processed by using the Maxwell Viral Total Nucleic Acid Kit on a Maxwell MDX platform (Promega). The amount of purified DNA was determined with the Qubit 4 Fluorometer (ThermoFisher Scientific, Carlsbad, CA, USA) using a Qubit RNA BR Assay Kit (ThermoFisher Scientific). Quality control, libraries preparation, HTS on Illumina NovaSeq 6000 platform (paired-end mode, reads of 150 bps), and preliminary analyses were carried out by an external service (IGA Technology, Udine, Italy).

#### 2.7.2. RNA Sequencing

Collected samples were processed by using the Maxwell 16 SimplyRNA Cells Kit on a Maxwell MDx platform (Promega). The amount of purified RNA was determined with the Qubit 4 Fluorometer using a Qubit RNA BR Assay Kit (ThermoFisher Scientific), and RNA integrity was assessed by agarose gel electrophoresis. Quality control, rRNA depletion, libraries preparation, and HTS on Illumina NovaSeq 6000 platform (paired-end mode, reads of 150 bps) were carried out by an external service (IGA Technology, Udine, Italy).

#### 2.7.3. Sequence Data Processing

IGA returned FASTQ reads files for further data processing, conducted in our laboratory. The received FASTQ files containing the sequenced reads were examined with quality control checks performed with FastQC (v0.12.1). Trim Galore! (v0.6.7) was used to trim the sequencing adapters and filter out low-quality reads.

### 2.8. Viral Genome and Transcriptome Data Analysis

#### 2.8.1. DNA and RNA Alignment on Viral Genome

The alignment of both DNA and RNA reads of each sample to the Parvovirus B19 (B19V) EC Genotype I consensus sequence (GenBank KY940273.1) was performed with HISAT2 (v2.2.1) [20]. SAMtools (v1.22) [21] was then used to convert each generated SAM file into a BAM file before sorting and indexing.

#### 2.8.2. Sequence Strings Search

seqkit (v2.10.0) [22] has been utilized to search for specific sequence strings within the trimmed reads of the sequenced samples (Appendix A, Table A2). The files were then processed using seqkit’s grep subcommand to perform a case-insensitive search for the forward input sequence. This search yielded a collection of FASTQ files containing the sequence strings of interest. These sequences were aligned to the B19V genome, and the resulting BAM files were used to generate coverage graphs. The generation of these graphs was performed with a custom Python script (v3.12.11), which leverages the pysam (v0.23.3) library to read and process the indexed BAM files.

### 2.9. Cellular Transcriptome Data Analysis

#### 2.9.1. Transcript Quantification and Import

Transcript abundance was quantified from the quality-controlled FASTQ files using Salmon (v1.10.3) [23], which employs a pseudoalignment algorithm to estimate transcript-level counts. The reference human transcriptome utilized in the study was downloaded from GENCODE (release 48) [24]. The resulting quantification files of Salmon were imported into the R statistical environment using the tximport R package (v1.36.0) [25]. This process aggregates transcript-level counts to the gene level, correcting for potential changes in transcript length across samples. Transcript-to-gene mapping was based on the metadata downloaded from the same source. Transcripts lacking corresponding gene symbols in the GENCODE metadata file, approximately 30% of all transcripts, were excluded from downstream analyses as these primarily represent unannotated or poorly characterized transcripts.

#### 2.9.2. Statistical Design

To assess the temporal and infection-related dynamics of gene expression, a multifactorial linear model was employed, incorporating the variables of hours post-infection (hpi) and infection status, along with their interaction terms.

Within the distributional model of edgeR [26], the expected number of reads assigned to gene g in sample i, indicated as $\mu_{gi}$, is modelled as a log-linear model of the form

(1) $$\log\mu_{gi}=x_{i}^{⊺}\beta_{g}+\log L_{i}$$

where $x_{i}$ is a covariate vector specifying the experimental conditions applied to sample i, $\beta_{g}$ is the coefficient vector capturing the experimental effects and log-fold-changes, whilst $L_{i}$ is the effective library size of sample i.

The actual number of reads assigned to gene g in sample i is indicated as $y_{gi}$ and is expected to follow a mixture distribution across biological replicates with a quadratic mean–variance relationship of the form

(2) $$\mathrm{var}\left(y_{gi}\right)=\sigma_{g}^{2}\mu_{gi}+\psi_{g}\mu_{gi}^{2}$$

where $\sigma_{g}^{2}$ represents the technical variation, or quasi-dispersion, and $\psi_{g}$ represents the biological variation, also referred to as the biological coefficient of variation (BCV) [27].

This design enabled the interrogation of both the main effects (infection or time alone) and interaction effects (e.g., impact of infection over time) leading to gene dysregulation. Linear combinations of model coefficients, also referred to as contrasts, defining specific biological comparisons of interest were created using the limma R package (v3.64.1) [28] to analyze the effect of infection at each time point as well as the differential effect of infection across time points.

#### 2.9.3. Data Pre-Processing and Quality Control

The gene-level count matrix was used to create a DGEList object, the primary data structure of the edgeR package (v4.6.2) [26]. This object encapsulates the raw count data, sample grouping information, and the experimental design matrix. Genes with a low expression level across all samples were filtered out to reduce noise. Normalization was carried out using the Trimmed Mean of M-values (TMM) method to account for differences in library size between samples.

#### 2.9.4. Exploratory Data Analysis

Following pre-processing, exploratory data analysis was conducted to assess sample relationships and identify potential outliers or batch effects. Principal Component Analysis (PCA) was performed on the ${log}_{2}$-transformed Counts Per Million (CPM) gene counts using the edgeR’s cpm function, and the first two components were visualized with the factoextra package (v1.0.7), offering an overview on the dissimilarity in gene-level expression counts across all samples. Multidimensional Scaling (MDS) was performed on the raw gene counts and visualized using plotMDS from the edgeR package on the top 500 genes. In this plot, the distances between samples approximate the expression differences between samples. The BCV (Equation (2)) was evaluated via the BCV plot generated with plotBCV from the edgeR R package after applying the estimateGLMRobustDisp method [29], where the BCV for each gene is plotted against its average log_2_-transformed CPM. BCV measures how much the true abundance of a gene differs between replicates, that is, not due to technical errors and not diminishing with increasing gene counts. Although the BCV is expected to remain approximately constant with increasing gene count size, in reality it is subject to small variations. Low overall BCV values and a smooth trended dispersion line indicates good biological reproducibility and appropriate modeling of the mean–variance relationship in the model.

#### 2.9.5. Model Fitting

Differential gene expression was assessed using the robust quasi-likelihood negative binomial generalized log-linear model with the glmQLFit function from edgeR. Subsequently, the quasi-likelihood dispersion was plotted using the plotQLDisp function, in which the quarter-root of the quasi-likelihood dispersion for all genes, before and after shrinkage towards a trend, is compared to the gene abundance in ${log}_{2}$ counts per million. A tight clustering of the squeezed dispersion values towards the trend line indicates that the model accurately describes the data.

#### 2.9.6. Contrast Testing

Subsequently, statistical testing for individual contrasts was carried out under two conditions: (i) without applying a log-fold change (logFC) threshold using glmQLFTest, and (ii) with a logFC threshold of 1.6 using glmTreat. The stringent logFC threshold of 1.6 was applied to focus on genes with substantial dysregulation, as the research prioritized the identification of the most strongly affected biological pathways. Results from both conditions were processed using the decideTests function in edgeR, which classifies genes as upregulated, downregulated, or not significantly changed. For both conditions, a *p*-value < 0.05 was used as a threshold of significance. Volcano plots contrasting two different conditions to one another were created using the ggplot2 R package (v3.5.2), whilst heatmap and UpSet plot visualizations were created with the ComplexHeatmap R package (v2.24.0) [30].

#### 2.9.7. Gene Set Enrichment Analysis

Competitive gene set testing was performed using the Camera [31] method implemented in edgeR, evaluating the enrichment of genes across the MSigDB hallmark collection accounting for inter-gene correlation [32]. This analysis evaluated the enrichment of dysregulated gene sets associated with curated biological pathways and conditions within the experimental data. Lollipop plots visualizing the direction of dysregulated gene sets, as well as the percentage of dysregulated genes in each set, was created using ggplot2.

### 2.10. Data Availability

Raw FASTQ reads were submitted to the European Nucleotide Archive, Accession PRJEB105312.

## 3. Results

### 3.1. Phenotypic Characterization of Erythroid Progenitor Cells

Erythroid progenitor cells (EPCs) were generated in vitro from peripheral blood mononuclear cells (PBMC), as described [16]. PBMC-derived cell cultures were grown until day 8 of differentiation, then characterized by flow cytometry for the expression of common cell surface markers of the erythroid lineage cells (CD36, CD71, GPA) and for the presence of the specific VP1u receptor (VP1uR) [18].

**Figure 2:**
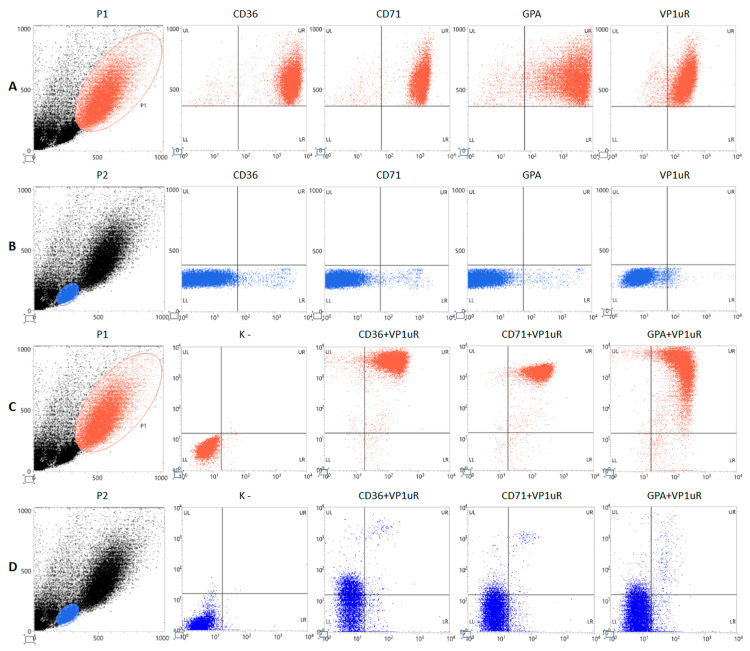
Morphological and phenotypical characterization of EPCs on day 8 of in vitro differentiation. Gating of subpopulations P1 (red dots) (**A**,**C**) and P2 (blue dots) (**B**,**D**); FSC, *X*-Axis; SSC, *Y*-Axis. (**A**,**B**): staining for erythroid differentiation markers (CD36, CD71, and GPA) (PE label, *X*-Axis; FSC, *Y*-Axis); staining for VP1uR via binding of VP1u (AF488 Label, *X*-Axis; FSC, *Y*-Axis). (**C**,**D**): negative control and double staining for VP1uR (AF488 Label, *X* Axis) and erythroid differentiation markers (CD36, CD71, GPA) (PE label, *Y*-axis).

By cytofluorimetric analysis (Figure 2, Table 2), the morphological parameters FSC and SSC highlighted the heterogeneity of the primary cell culture. A subpopulation gated in P1, counting 35% of cells, was selected for highest size and complexity. Close to 100% of the cells gated in P1 were positive for the erythroid lineage surface markers CD36, CD71, and GPA, with a high mean fluorescence intensity (MFI—geometric mean). This same subpopulation expressed VP1uR, also close to 100% frequency. As a term of comparison, a subpopulation of lower size and complexity, gated in P2 and counting 30% of cells, showed very low expression, by frequency and fluorescence intensity, of all the erythroid lineage markers and VP1uR. Further, double staining for either CD36, CD71, or GPA, each combined with VP1uR, showed that the subpopulation in P1 expresses VP1uR in close association with CD36, CD71, and GPA; this association is negligible for the subpopulation in P2. Altogether, data indicate the subpopulation in P1 as mainly composed of erythroid progenitor cells and the target for B19V infection, while the subpopulation in P2 resulted mainly composed of lymphocytes (Appendix A, Table A1).

### 3.2. Course of Infection of B19V in EPCs

EPCs on day 8 of in vitro differentiation were infected with B19V at a moi of 10^3^ genome copies/cell and were then sampled at 2, 16, and 48 hours post-infection (hpi). Investigation of B19V replication was first carried out by Flow-FISH and quantitative molecular analysis.

**Figure 3:**
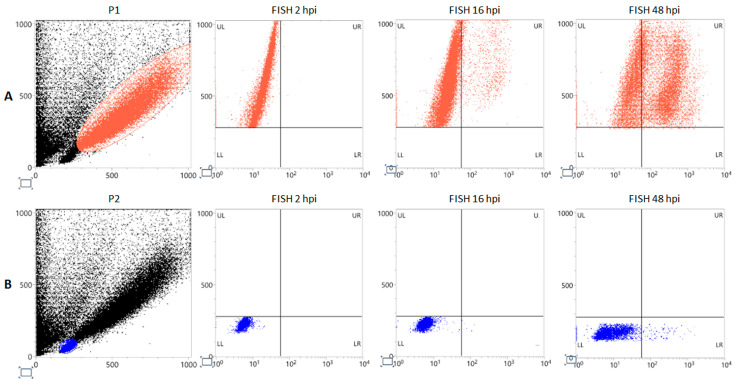
Flow-FISH for viral nucleic acids in infected EPCs. Morphological characterization of EPCs at day 8 of in vitro differentiation and gating of subpopulations P1 (red dots) (**A**) and P2 (blue dots); (**B**) at 2 hpi 16 hpi, 48 hpi; FITC labeling, *X*-Axis. FSC, *Y*-Axis.

Flow-FISH (Figure 3) identified the subset of cell population permissive to viral replication. Distribution of positive signal indicated cells corresponding to the P1 subpopulation as the only supporting a productive replication, showing 5.1% positivity at 16 hpi, rising up to 54.2% at 48 hpi, compared to the P2 subpopulation which showed 0.1% and 1.2% positivity at 16 hpi and 48 hpi, respectively.

**Figure 4:**
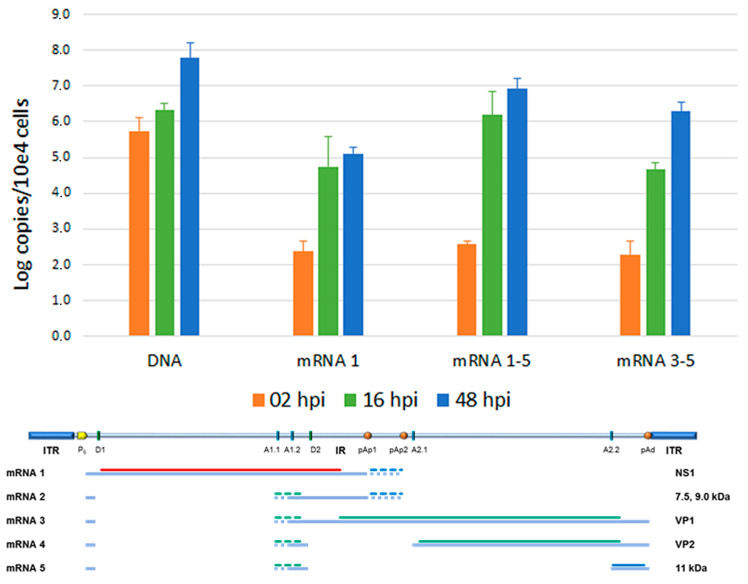
Quantitative analysis of viral genomic DNA, total viral mRNA, and mRNA subsets in EPCs at the indicated time points. Quantitative data obtained from three independent replicate experiments, each determination in duplicate; bars indicate mean + SD.

Total nucleic acids were then investigated by qPCR analysis, to quantitate the variation in the amount of viral DNA, and qRT-PCR, to quantitate the variation in the amount of total mRNA via the central exon (mRNA 1–5) and of specific subsets of viral mRNAs, particularly the unspliced mRNAs (mRNA 1) or the distally cleaved mRNAs (mRNA 3–5) (Figure 4). Viral DNA, starting from a 5.73 Log B19V genome copy at 2 hpi (per 10^4^ cells, ~7% of viral input), showed a +0.59 Log variation from 2 to 16 hpi, reaching +2.04 Log at 48 hpi, confirming a productive infection pattern. Total viral mRNA, already detectable at 2 hpi (2.56 Log), showed a +3.62 Log variation at 16 hpi and a +4.36 Log variation at 48 hpi, also confirming the productive pattern of infection. The majority of mRNAs were constituted by the spliced, proximally cleaved mRNAs (mRNA 2); unspliced, proximally cleaved mRNAs (mRNA1) coding for the NS1 protein were present at 5.5% and 1.4% abundance at 16 hpi and 48 hpi, respectively; and the distally cleaved mRNAs (mRNA 3–5), coding for the VP1, VP2 and 11 kDa proteins, were present at 1.5% and 22.7% abundance at 16 hpi and 48 hpi, respectively. This pattern is in accordance with the known expression profile of B19V, at early and late times of infection, and in relation to the replication of the viral genome.

To further characterize the cell population supporting B19V replication, EPCs at day 8 were sorted by FACS, selected for the presence of the VP1uR. The initial not sorted, sorted and positively selected, and sorted and negatively selected cell populations were then infected, and the outcome of infection was monitored by qPCR. Both the not-sorted and the sorted–positive cells supported B19V replication to the same extent, while replication did not occur in the sorted–negative cells. Thus, only the erythroid cells, corresponding to the P1 population, were permissive to viral replication; the presence of other cell types, including lymphocytes in P2 population, did not exert any relevant restriction to viral replication (shown in Supplemental File S1).

### 3.3. HTS Analysis: B19V Genome in EPCs

**Figure 5:**
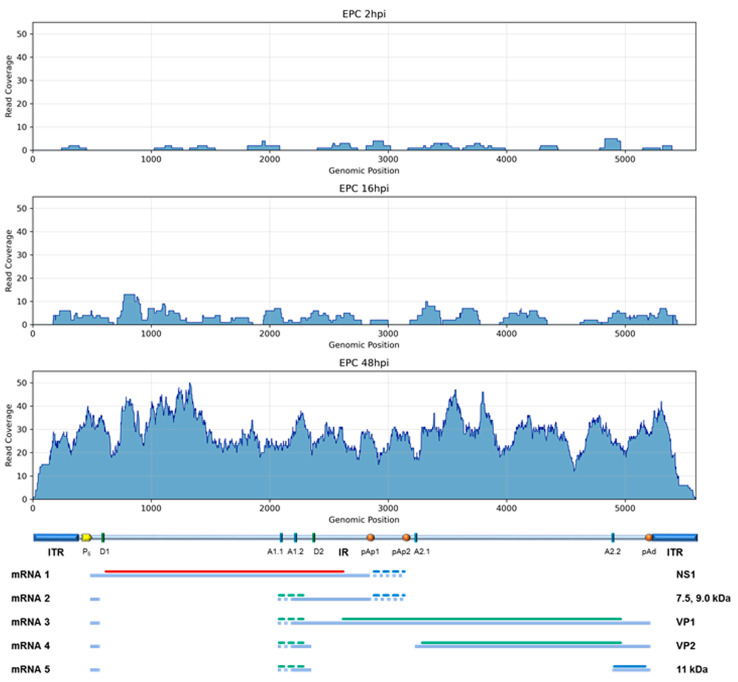
Plot of coverage of HTS DNA reads, aligned on B19V genome at 2, 16, and 48 hpi.

To identify B19V genome within total genomic HTS data, processed reads were selected and aligned on the reference EC B19V genome (GenBank: KY940273.1) (Figure 5). At 2, 16, and 48 hpi, 0.003%, 0.007%, and 0.09% of reads mapped on the viral genome, respectively. In particular, at 48 hpi, mapping showed a continuous coverage over the whole genome length, with an average depth of reading of 28.4 counts per base; the amount of reads mapping on B19V, although low in absolute, represent a ~10^3^ molar excess of B19V with respect to the cellular genome, in good agreement with data obtained by qPCR. With respect to the viral genome, no major deletions, rearrangements or recombination events were observed; additionally, viral–cell junctions hinting at integration events were not detected.

### 3.4. HTS Analysis: B19V Transcriptome in EPCs

**Figure 6:**
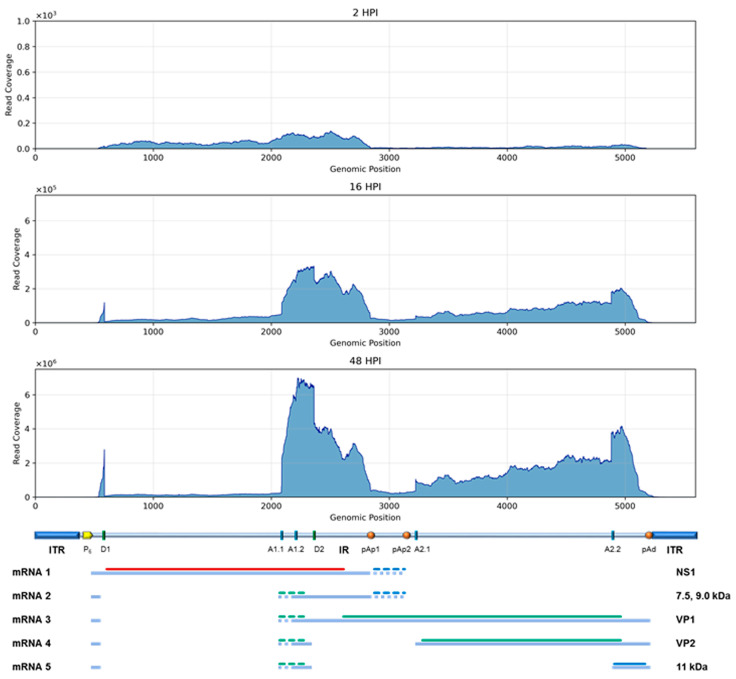
Plot of coverage of HTS RNA reads, aligned on the B19V genome at 2, 16, and 48 hpi.

To identify B19V mRNAs within total transcriptomic HTS data, processed reads were selected and aligned on the reference EC B19V genome (Figure 6). Reads mapping on the viral genome constituted a barely detectable 0.001% at 2 hpi, increasing to 1.8% at 16 hpi and reaching 24% at 48 hpi. The identification of viral reads and their alignment on the B19V genome, significative at 16 hpi and especially at 48 hpi, allowed for tentative reconstruction of the viral transcriptome. The different regions of the viral genome were differentially represented in relation to the pre-mRNA processing events and generation of mature mRNAs, showing good correspondence to the known transcription map of B19V; the few reads identified at 2 hpi mapped on the left side of the genome, in correspondence with the NS1 coding region, while the reads mapped at 16 and 48 hpi were mostly in the central exon region and on the right side of the genome, in correspondence with VP1/2 and 11 kDa coding regions. However, a systematic bias was observed in the coverage, resulting in abundance gradients from the center to the extremities of represented exons. Thus, by normalizing the number of reads mapped to the different genomic regions to the respective length in bases, a fractional distribution was obtained confirming a higher relative representation of the leader and central exons, compared to the regions coding for NS and VP proteins and to the terminal exon (Table 3).

**Figure 7:**
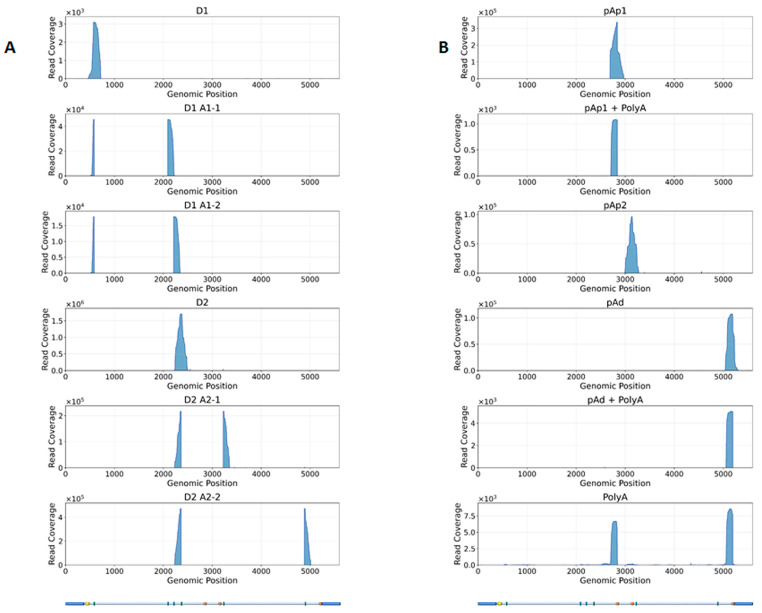
Plot of coverage of selected RNA reads aligned on B19V genome at 48 hpi. Reads discriminate the differential splicing (**Panel A**) and cleavage-poly-A patterns (**Panel B**).

To obtain a more accurate evaluation of the relative abundance of the different viral mRNAs, a complementary strategy was followed, aimed at determining the frequency of alternative splicing and cleavage-polyadenylation processing patterns. For this purpose, all reads mapping to the B19V genome and spanning the reported splice junctions and cleavage-polyadenylation sites (Appendix A, Table A2) were specifically selected, mapped, and quantitated (Figure 7). This closer inspection of mapping involved the known mRNA processing sites: (1) start of transcription at nt 530; (2) donor/acceptor splice sites at 586/2089–2209 (D1/A1.1–A1.2); donor/acceptor splice sites at 2363/3224–4883 (D2/A2.1–A2.2); the pAp1 cleavage-polyadenylation site at 2842, and the pAp2 site at 3142; and the pAd distal cleavage polyadenylation site at 5189. The frequency of utilization of the different signals was reconstructed by the relative frequency of reads mapped at each selected site (Table 4).

Concerning the splicing processing sites, the first intron showed a prevalence of splicing events already at 16 hpi, 8% unspliced vs. 92% spliced (65% D1–A1.1, 27% D1–A1.2), still increasing at 48 hpi, 3% unspliced vs. 97% spliced (77% D1–A1.1, 20% D1–A1.2). In opposition, the second intron showed a lower splicing frequency both at 16 hpi, 69% unspliced vs. 31% spliced (5% D2–A2.1, 26% D2–A2.2), and at 48 hpi, 61% unspliced vs. 39% spliced (9% D2–A2.1, 30% D2–A2.2).

Concerning the cleavage-polyadenylation sites, investigation was severely biased by the steep decrease in reads coverage towards the mRNA 3′ ends, and results cannot account for the total amount of processed mRNA. Only considering the number of reads mapped across the known poly-A cleavage sites, ratios of cleaved (poly-A containing) to un-cleaved (readthrough) mRNAs, were 38% at 16 hpi to 48% at 48 hpi for pAp1; 7% to 6% for pAp2; and 60% to 28% for pAd. Direct inspection for poly-A containing reads across the whole genome found a low number of reads, selectively identifying the pAp1, but not pAp2, and the pAd-processed mRNAs.

### 3.5. HTS Analysis: Cellular Transcriptome Dynamics

For both controls and virus-infected cell cultures, triplicate samples were collected at each time point. Transcript abundance was quantified with the pseudo-alignment algorithm of Salmon against the human transcriptome (GENCODE, release 48). Groups were compared as either synchronic (infected against not-infected at the same time point) or diachronic (infected against not-infected across time points). Synchronic comparisons revealed virus-induced changes in cellular gene expression profiles, while diachronic comparisons captured changes in cellular gene expression during in vitro growth and development under viral influence. Data exploration was then performed to assess the discriminatory power and overall quality of the experimental data.

**Figure 8:**
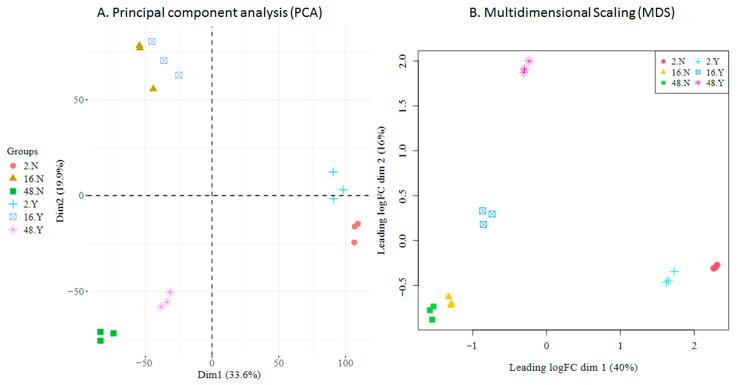
HTS cellular transcriptome; data exploration. (**A**). Principal Component Analysis (PCA); the two main dimensions account for 53% of total variation. (**B**). Multidimensional Scaling (MDS); the two main dimensions account for 56% of total variation. Groups are defined as hpi (2, 16, 48), not-infected or infected (N, Y), and replicate (1–3).

Principal Component Analysis (PCA) (Figure 8A) revealed that samples primarily clustered by hours post-infection (2 hpi, 16 hpi, 48 hpi), with viral infection providing a secondary separation. This pattern indicates that ongoing in vitro differentiation is the dominant driver of cellular transcriptomic variation in the experimental setting, while viral influence modulates transcriptomic variation, an effect more prominent at 2 hpi and 48 hpi compared to 16 hpi. Within each condition, replicate variability was minimal, underscoring the high consistency and reliability of the data. Multidimensional Scaling (MDS) (Figure 8B) applied to the top 500 genes revealed a similar separation pattern between samples; although the effect of the infection status was more prominent in this plot, replicates clustered together, reaffirming the consistency of the data.

**Figure 9:**
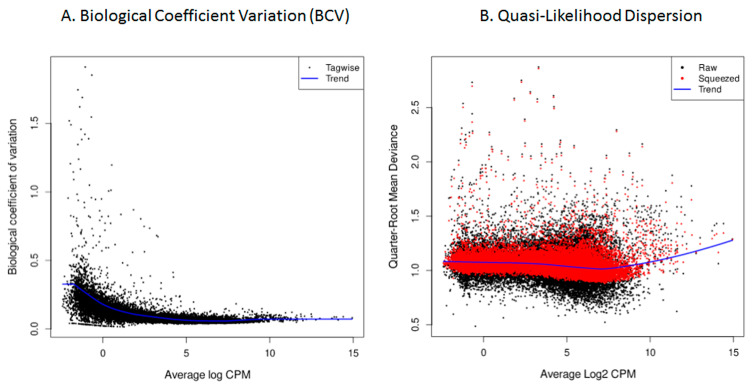
HTS cellular transcriptome; data quality assessment. (**A**). Biological Coefficient Variation (BCV), in which the *X*-axis depicts the mean genetic expression in log count per million (log CPM), while the *Y*-axis represents the biological coefficient of variation. (**B**). Quasi-likelihood dispersion: quarter-Root mean deviance plotted against Log2-transformed CPM.

As a quality assessment, gene count dispersion was visualized using a Biological Coefficient Variation (BCV) plot. This coefficient estimates the irreducible variation in true gene abundance between replicates. In the analyzed samples, BCV values were low, allowing for a better distinction of differentially expressed genes (DEGs) with higher reliability (Figure 9A). After fitting the model with the *glmQLFit* function of *edgeR*, quasi-likelihood dispersion (Equation (2)) was visualized using *plotQLDisp*. The tight clustering of the squeezed data points around a relatively stable trend line across expression levels indicates that the quasi-likelihood dispersions was well estimated and that the variance structure was appropriately captured by the model (Figure 9B).

**Figure 10:**
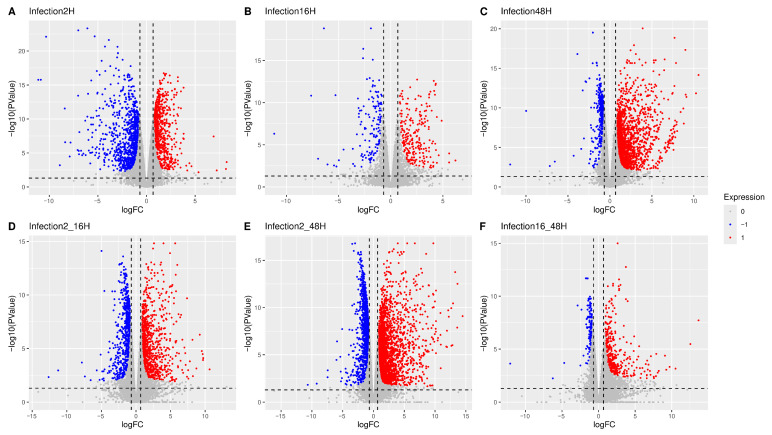
In volcano plots, the *X*-axis represents the logFC whilst the *Y*-axis represents the negative logarithmic *p*-value. Each point in the plot represents a gene; the color indicates the predicted regulation (red for upregulation, blue for downregulation, and gray for non-significance). Significance was tested for an absolute log fold-change greater or equal to 1.6 for synchronic (**A**–**C**) or diachronic (**D**–**F**) contrasts. A complete list of genes is in Supplemental File S2.

Differential Gene Expression Analysis (DGEA) was carried out on the samples according to the study design. At the whole population level, this analysis identified numerous dysregulated genes across the different experimental contrasts. Genes exhibiting a significant differential expression were visualized as volcano plots between paired conditions (Figure 10). Specifically, considering synchronic contrasts (A–C), samples at 2 hpi (A) showed predominantly downregulated cellular genes, 16 hpi (B) displayed fewer and more balanced changes, while 48 hpi (C) was characterized mainly by upregulated genes. Diachronic contrasts (D–F) variation was more pronounced at the early stage of infection (2–16 hpi, D) than at later stages (16–48 hpi, F). Across the overall infection time course (2–48 hpi, E), upregulation of cellular genes was prevalent over downregulation.

**Figure 11:**
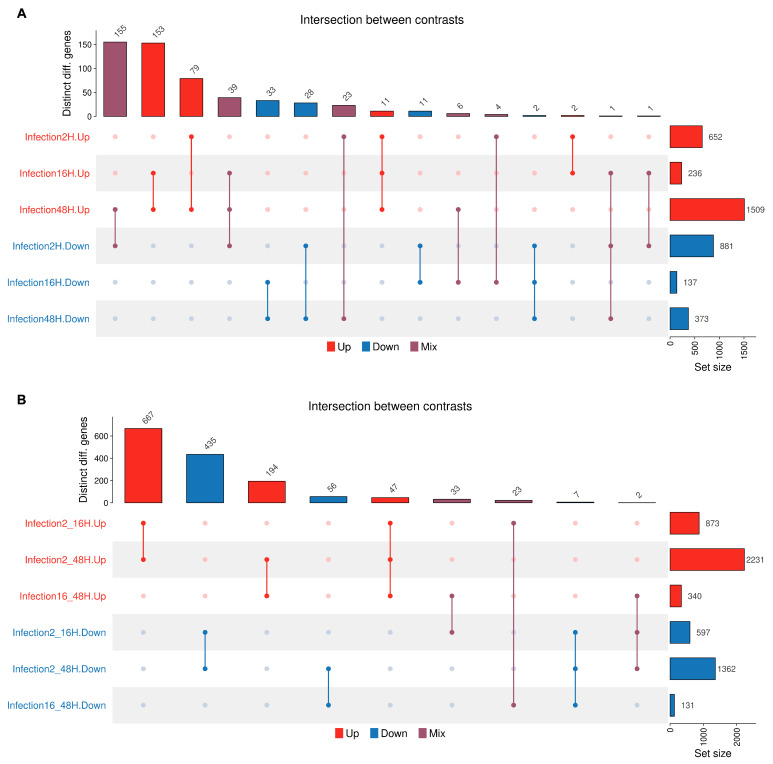
The UpSet plots visualize the set size and intersection of dysregulated genes across different comparisons, either upregulated (red bars), downregulated (blue bars), or contrasting (purple bars). Rows, number of genes within each comparison group; columns, number of genes at intersections of the different comparison groups. (**A**): Synchronic contrasts; (**B**): diachronic contrasts.

The UpSet plot (Figure 11) visualizes the size of intersections among the sets of differentially expressed genes identified across synchronic or diachronic contrasts under the specified experimental conditions. In synchronic contrasts (A), gene expression dysregulation is predominantly characterized by downregulation at the early stage of infection (2 hpi) and by upregulation during the later stages (48 hpi). The intersections, representing genes dysregulated across contrasts, involve smaller subsets of genes. Of these, the largest intersections include genes downregulated at 2 hpi and upregulated at 48 hpi, and genes upregulated both at 16 and 48 hpi. In diachronic contrasts (B), the largest intersections include genes constantly dysregulated from the early (2–16 hpi) to the whole (2–48 hpi) time course of infection.

**Figure 12:**
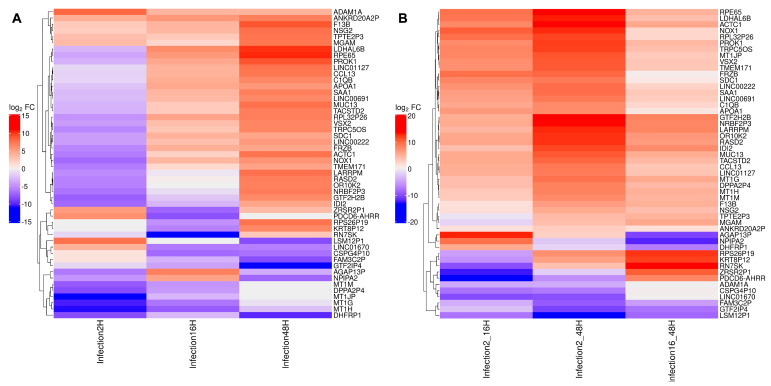
The top 50 differentially expressed genes identified across the synchronic (**A**) and diachronic (**B**) contrasts are displayed in a heatmap. Each cell corresponds to the logFC of a given gene within a specific contrast, with colors ranging from red (upregulation) to blue (downregulation). The dendrogram on the left groups genes into clusters based on the similarity of their logFC profiles.

By selecting the 50 mostly differentially expressed genes across synchronic or diachronic contrast, signatures can be recognized, differentiating the cellular transcriptomic profile based on cluster analysis (Figure 12). In particular, in the synchronic comparison, four major groups include (i) genes mostly downregulated at 2 hpi; (ii) genes mostly downregulated at 16 and 48 hpi; (iii) a large set of genes downregulated at 2 hpi but upregulated at 16 and 48 hpi; and (iv) genes constantly upregulated. In the diachronic comparison, four groups include genes constantly downregulated from 2 to 48 hpi, genes with contrasting regulation at early (2–16 hpi) compared to late (16–48 hpi) times, and, the largest group, genes constantly upregulated over the whole course of infection.

### 3.6. HTS Analysis: Cellular Transcriptome Enrichment Analysis

**Figure 13:**
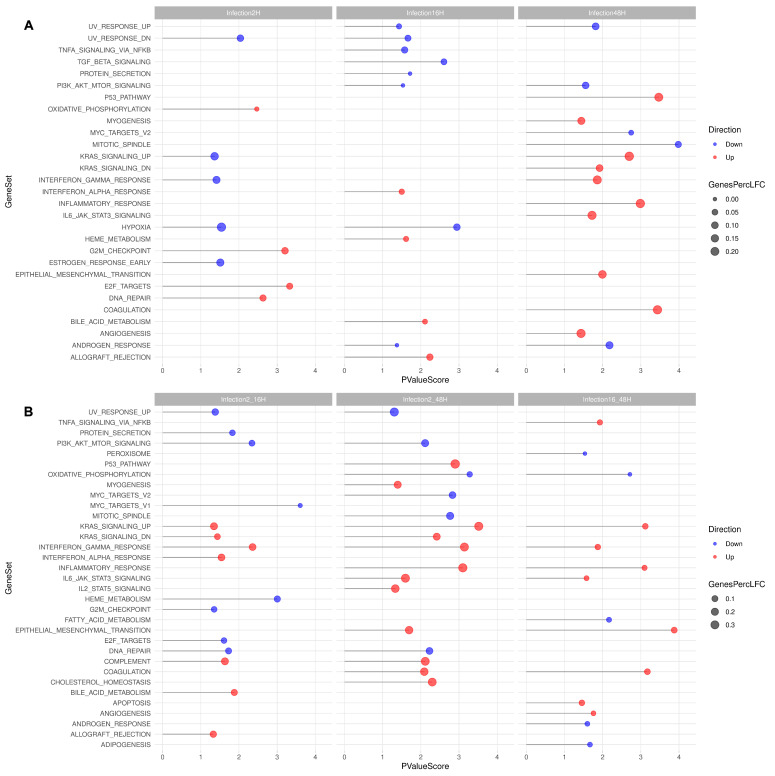
GSEA. Inside the hallmark collection of MSigDB, several pathways were found to be enriched in the analyzed samples, considering synchronic (**A**) or diachronic (**B**) contrasts. In the lollipop plot, the *X*-axis reflects the negative logarithmic *p*-value, the color of the dot reflects either up- (red) or downregulation (blue). The size of the dot reflects the amounts of genes found in both the analyzed samples and the tested gene set. A complete list of genes is in Supplemental File S3 for synchronic contrasts and Supplemental File S4 for diachronic contrasts.

To characterize expression patterns, gene set enrichment analysis (GSEA) was performed using Camera on the Molecular Signature Database (MSigDB) Hallmark gene set collection [32]. Of the 50 sets listed in MSigDB, GSEA performed across all the different experimental contrasts selected 37 sets, including 3203 genes. Following scrutiny for significant LogFC, analysis yielded a restricted group of 798 genes (Figure 13).

When investigating synchronic contrasts (A), GSEA selected 29 sets, including 2729 genes, restricted to 457 following scrutiny for significant LogFC. For the different time points post-infection, downregulation was prevalent at 2 hpi (92 downregulated vs. 57 upregulated genes), while upregulation was prevalent at 48 hpi (66 downregulated vs. 234 upregulated genes). Of interest and higher statistical value, at 2 hpi, an upregulation of DNA repair, E2F targets, G2M checkpoint was noted; at 2 hpi, a downregulation of hypoxia response was noted; and at 48 hpi, an upregulation of the p53 pathway, Kras signaling, Interferon gamma response, inflammatory response, and IL6_Jak_STAT3_signaling, coagulation was noted.

When investigating diachronic contrasts (B), GSEA selected 33 sets, including 2988 genes, restricted to 654 following scrutiny for significant LogFC. In particular, upregulation was prevalent over the whole course of infection, from 2 to 48 hpi (163 downregulated vs. 453 upregulated genes). Of interest and higher statistical value, considering the 2–48 hpi time course, upregulation of the p53 pathway, Kras signaling, Interferon gamma response, inflammatory response, IL6_Jak_STAT3_signaling, IL2_STAT5 signaling, complement, coagulation, and cholesterol homeostasis was noted.

### 3.7. Interaction Networks

Exploration of interaction networks was conducted by exploiting the STRING database [33]. Gene sets previously identified were provided as query terms, and a clustered analysis with the Markov Cluster Algorithm (MCL) was performed. Results are shown in Appendix A, Table A3 for the most relevant clusters, according to samples (synchronic or diachronic contrasts). A full list of clusters and related genes is presented in Supplemental File S5. For all investigated gene sets, STRING analysis returned significantly enriched interaction networks, differing in terms of abundance and composition. Clusters were interpreted in terms of Reactome pathway database [34], highlighting different biological processes differentially affected in the course of infection and possible key nodes within each one.

At 2 hpi, downregulation was prominent. A major cluster involved generic signal transduction and gene transcription pathways, particularly related to IL-4 and IL-13 cytokine signaling, and cell cycle-related pathways, particularly the G1/S checkpoint, with a nodal position for cyclins CCND1, CDKN2C, CCNE1, CDKN1B, and MYC. A large cluster also involved generic signal transduction and PERK regulated gene expression, mostly related to the AP-1 family of transcription factors, with JUN and FOS as central nodes. An additional cluster involved innate immune system response, CCL2. At 2 hpi, upregulation mainly involved genes related to cell cycle and DNA repair pathways, key nodes AURKA and FEN1, and Interferon alpha/beta signaling, including OAS2.

At 16 hpi, the impact on cell transcriptome was less prominent on the cell cycle, while shifting towards an emerging immune response. Upregulated gene sets were related to extracellular matrix organization, cytokine signaling, and MHC class II antigen presentation. Downregulated gene sets involved oxidative stress response and response to metal ions.

The shift in gene expression patterns towards an activating immune response was prominent at 48 hpi. A large gene set encompassed upregulation of genes involved in innate as well as adaptive immune response. The most interconnected network included genes involved in inflammatory-type response, with TNF, IL1B, CCL2, and CCL5 playing a central role; genes involved in inflammatory response via FPR1 and TLRs; genes involved in MHC-II antigen presentation and Interferon gamma pathways such as CD74, IFI30, HLA-B, and HLA-DMA; and genes involved in Interferon alpha/beta signaling, such as IFITM1, XAF1, SAMHD1, and ISG20. Additional smaller networks emerged, included platelet degranulation and detoxification of Reactive Oxygen Species. Downregulation at 48 hpi was less prominent, mainly affecting genes involved in response to heme and aminoacid deficiency.

When considering the diachronic gene expression variation in the course of infection, from 2 to 48 hpi, the genes involved constituted a highly interconnected network, mostly composed of upregulated gene sets. These included a large set of genes involved in the innate immune response through cytokine signaling; a set of genes involved in the adaptive immune response, through upregulation of MHC-II antigen presentation; a further set of genes involved in Interferon alpha/beta signaling; the rescue, compared to the prevalent downregulation at 2 hpi, of signal transduction and transcription pathways connected to AP-1 and Myc; and genes connected to extracellular matrix organization and to platelets activation. The most prominent downregulation concerned genes relevant to the mitotic phase of the cell cycle, which were prominently upregulated at 2 hpi.

Thus, to summarize variations in gene expression levels within a highly complex interaction network, a common theme is the transition from an early antiviral state, with cell cycle deregulation and shut-off of cellular transcription activity, to an immune response evolving from innate, mainly inflammatory character, and to an adaptive response, with a peculiar upregulation of genes involved in antigen processing and MHC-II presentation.

## 4. Discussion

Erythroid progenitor cells are the main target of B19V, selectively constituting a cell population both susceptible and permissive to productive viral replication. In turn, dysregulation of erythropoiesis and bone marrow environment caused by B19V infection is a crucial aspect of B19V-related pathogenesis. The availability of PBMC-derived, in vitro differentiated EPCs offers the appropriate experimental setting for studying virus–cell interactions and constitutes a suitable model to study the impact of viral infection on this cell population [14,15,16]. In our work, in addition to phenotypical investigation by cytofluorimetric techniques, and direct quantitative assessment of viral replication by molecular techniques, high-throughput sequencing and a refined bioinformatic pipeline allowed us to investigate virus–cell interactions in a comprehensive, systematic approach.

PBMC-derived EPC cell cultures, however, constitute a heterogenous cellular population, characterized by the compresence of multiple differentiation trajectories and developmental stages, providing a very complex cellular landscape [35,36,37]. On day 8 of in vitro culture, only a subpopulation (P1), about 35% of cultured cells, could be identified as differentiated erythroid progenitors given the distribution of specific markers such as CD36, CD71, and GPA. Importantly, only this subpopulation showed the concomitant presence of the cellular receptor for the VP1u domain, a key determinant of viral tropism (Figure 2). Cells positive by flow-FISH analysis, as a marker of a productive infection, were confined within the P1 population (Figure 3), at increasing amounts from 5% at 16 hpi to about 50% at 48 hpi. By quantitative molecular analysis, a productive infection within the cell population was confirmed at 48 hpi, yielding about 10^8^ genome copies per 10^4^ cells, a net increase of 2 Log compared to input virus at 2 hpi, and about 10^7^ mRNA per 10^4^ cells, a net increase of 4 Log from 2 hpi to 48 hpi (Figure 4). In addition to differentiated erythroid progenitors, cell cultures were characterized by the presence of a subpopulation (P2), about 30% of cultured cells, mainly positive to CD45 antigen and thus identified as lymphocytes. While not susceptible or permissive to viral replication, this population is expected to contribute to cell population dynamics in response to viral infection.

A productive replication and complete expression pattern of B19V expression were thus confirmed in the presented experimental setting, restricted to a subset of cells characterized by a mature erythroid phenotype, the presence of the VP1uR, but by an ununiform permissive intracellular environment. This information constitutes a framework for interpretation of the study of the dynamics of B19V replication and its impact on the cell population by means of high-throughput sequencing (HTS). HTS was aimed at retrieving information on the presence and amount of B19V DNA, of B19V transcripts, and on the variation in the cell population transcriptome as a consequence of B19V infection. Since HTS was carried out for the total cell population, all information obtained refers to average quantities measured over the whole population rather than to their distribution. Due to the heterogenous characteristic of EPC population and the observed restrictive pattern of permissiveness to B19V, this is a limitation that needs to be considered.

By HTS, the viral DNA genome could be identified by selection and alignment of reads obtained from whole-genome sequencing; at 48 hpi, about 0.1% of total reads mapped to B19V genome, reaching 100% coverage with an average depth of about 30 counts per position. Given the direct sequencing without prior selective amplification, the reads likely reflect the presence of dsDNA replicative intermediates; coverage is uniform along the viral genome, excluding the presence of major defective forms. The limited sequencing depth, however, prevents the investigation of sequence variability as a result of viral replication and any appraisal of a quasi-species structure of the viral population. To this purpose, dedicated experimental workflows are required [38,39].

HTS yielded more information in the analysis of the viral transcriptome. First, the amount of reads mapping on the viral sequence increased in the course of infection, reaching up to 24% at 48 hpi; considering the restrictive nature of infection, this figure implies prevalent commitment of productively infected cells in the synthesis of viral mRNAs. Then, mapping of reads showed good correspondence to the known transcription map of B19V, both in terms of localization and of relative abundance, reflecting the differential expression profile of B19V among early or late times in the course of infection. However, a limitation of the experimental approach emerged as an ununiform depth of coverage along the different regions composing the mature viral transcripts. This effect produced a decreasing abundance gradient from the center towards the extremities of transcripts and, also because of the complex transcription map of B19V, prevented a simple quantitative evaluation of the abundance of the different viral mRNAs based on differential read counting. Though, by normalization of reads coverage to genome region length, a quantitative evaluation was obtained, with good correspondence to what expected from quantitative molecular analysis. Specific information on the frequency of pre-mRNA processing events was sought by selecting reads selectively mapping on splicing or cleavage/poly-A sites. This strategy was quite successful for investigation of splicing events, yielding both an accurate mapping and a quantitative evaluation of the relative splicing patterns. Limitations, on the other hand, emerged for the investigation of the cleavage-polyadenylation events, since only a minority of reads actually identified the poly-A containing viral mRNAs; thus, an accurate evaluation of cleaved to readthrough transcript abundance over the different processing sites has likely not been achieved.

Analysis of the cellular transcriptome and its variation over the course of infection was a relevant outcome of HTS and downstream bioinformatic analysis. Observed variation arose due to two concurrent processes, namely the differentiation of the progenitor cells along the erythropoietic process and the virus infection, which impacted the transcription profile observed at each given time point and deviated the trajectory of the cellular transcription profile. Hence, it emerged the necessity to incorporate in the analytical model both sources of variations and the performance of both synchronic (same time points) and diachronic (different time points) comparisons of infected cells vs. not-infected controls. The analysis of RNA sequencing data was then carried out to identify differentially expressed genes between contrasting experimental groups, particularly to identify high-confidence DEGs, and to perform gene set enrichment analyses to uncover the main biological processes affected by the infection.

Overall, results indicate that variation in the transcriptome of EPCs is mainly driven by the ongoing differentiation process, with the virus impacting to a lesser level, but still clearly separating infected vs. non-infected profiles. Considering synchronic comparisons, differences in the expression profile were more evident at 2 hpi and 48 hpi, and, to a lesser extent, at 16 hpi. This scenario is supported by the diachronic comparison at early (2–16 hpi) compared to late (16–48 hpi) times post-infection also showing distinct patterns, as well as by the comparison over the whole time course of infection that clearly separated sets of down- to upregulated genes.

Gene set enrichment analysis was carried out on the differentially expressed genes as a means of evaluating the impact of the virus in the cell population. In a very complex transcriptional landscape, with different cell types contributing to different extents, some emerging features can be discerned. At 2 hpi, before any significant onset of viral protein synthesis, variations were likely induced by the cellular sensing of viral infection; involved pathways included DNA damage repair, regulation of cell cycle at G1/S checkpoint, and the AP-1 related transcription network. Overall, these processes should be regarded as the induction of an antiviral state within infected cells, whereas the virus may in turn exploit these mechanisms at its own advantage. As a working hypothesis, an ununiform distribution of processes in the cell population may determine the final balance in terms of permissiveness and explain the restrictive pattern of viral infection. At intermediate, 16 hpi, and late, 48 hpi, times post-infection, variations may arise from maturation of the initial cellular response, as well by the more direct effect of viral proteins, possibly segregated depending on the permissiveness to viral replication of the different cells. Related to the specific impact of viral proteins on cell environment, NS1 protein, as the major viral functional protein, is credited with many activities (reviewed in [6]), including induction of a DNA damage response, cell cycle arrest at both G1/S and G2/M, and induction of apoptosis and trans-activation of proinflammatory genes. A key role has been assigned to the interaction of NS1 protein with members of the E2F family, finely tuning the balance of activating or inhibiting pathways to regulate blocking of cell cycle progression and cell differentiation as a clue to B19V pathogenesis [40]. Such activities can be exerted either through dysregulation of gene expression networks or just by modulation of signaling pathways, without a direct effect on gene expression levels. Not all of these reported effects could be traced in our system, but considering our HTS data originating from a complex and dynamic system, unraveling the specific contribution of viral proteins to the variation in cell expression profiles will be an intense field of research.

Concerning the cellular response, the most striking trajectory indicates that the cellular population operates towards the induction of an inflammatory environment, mostly related to TNF and IL-10, and a subsequent transition to adaptive immunity with evidence of upregulation of genes involved in MHC-II presentation. In fact, PBMC-derived EPCs are endowed with immunoregulatory activity and are reported to express MHC-II complex [35,41]; viral infection may be causal to their observed upregulation and contribute significantly to the capacity of the immune response to contrast infection. Given the presence of a substantial lymphocyte population in the EPC culture, our experimental setting does not allow us to definitely assign antigen processing and presentation activity to cells in the erythroid lineage, leaving it as a hypothesis to be specifically tested. Some interplay is, however, expected between erythroid progenitors and lymphocytes, whose characterization will also be of relevant interest.

Given the complexity of the system, several sources of variability need to be considered in the interpretation of data to generate a paradigmatic model for B19V infection in EPCs. Variability is minimized for the virus, since a laboratory strain was used, whose sequence is a consensus sequence averaging with respect to single variants, and avoiding any interference arising from a source biological matrix such as patients’ plasma. A major source of variability is linked to the heterogeneity of the cell population as a result of its in vitro differentiation process and compresence of both erythroid progenitors and non-erythroid cells; therefore, the actual proportion of cells susceptible and possibly permissive to viral infection compared to bystander cells can be variable. In the presented set of experiments, the degree of both cellular differentiation and viral replication were in the expected range for PBMC-derived EPCs cell cultures, so results of HTS may also be considered representative; however, differences in different contests are to be anticipated. Finally, variability may arise from the genetic background of the host, especially if antigen processing activity is considered, and may contribute significantly to the outcome of the infectious process and cellular response.

## 5. Conclusions

As a result of our present work, a characterization of both viral and cellular expression profile in the course of infection of PBMC-derived EPCs was obtained, reconstructing the viral transcriptome and highlighting viral-induced variations in the cellular transcriptome. Given the heterogeneity of cell population, the difference in susceptibility to virus infection, the different patterns of expression of the viral genome, and the cellular response are supposed to also be highly heterogeneous. The reported experiments provide a comprehensive analysis of modulation of expression profile in the whole cell population, but single-cell analysis emerge as a necessary further step to fully elucidate the complex virus–cell relationship and the impact of virus infection on the physiology of erythroid cells [42]. In turn, this will allow better comprehension of the pathogenetic mechanisms of infection when translated on the bone marrow environment and, finally, a better definition of potential antiviral strategies.

## Figures and Tables

**Table 1:** Primer pairs used for qPCR and qRT-PCR analysis of B19V targets.

Primer	Sense	Primer	Antisense	DNA Target
18Sfor	CGGACAGGATTGACAGATTG	18Srev	TGCCAGAGTCTCGTTCGTTA	Genomic 18S rDNA
R2210	CGCCTGGAACACTGAAACCC	R2355	GAAACTGGTCTGCCAAAGGT	Virus DNA
**Primer**	**Sense**	**Primer**	**Antisense**	**RNA Target**
R1882	GCGGGAACACTACAACAACT	R2033	GTCCCAGCTTTGTGCATTAC	NS mRNA
R2210	CGCCTGGAACACTGAAACCC	R2355	GAAACTGGTCTGCCAAAGGT	Central exon, total RNA
R4899	ACACCACAGGCATGGATACG	R5014	TGGGCGTTTAGTTACGCATC	Distal exon, pAd cleaved

**Table 2:** Distribution of erythroid-specific markers in EPCs at day 8 of in vitro differentiation.

Population		CD36	CD71	GPA	VP1uR
P1	%	97.94	97.63	96.04	96.46
	MFI	3306	1353	2209	250
P2	%	2.99	2.38	1.85	2.08
	MFI	439	397	488	110
		**CD36 + VP1uR**	**CD71 + VP1uR**	**GPA + VP1uR**	
P1	%	96.10	97.87	94.91	
P2	%	2.33	1.28	1.85	

% of positive cells and relative MFI in subpopulations P1 and P2 as gated.

**Table 3:** Fractional distribution of HTS RNA reads on B19V transcription map.

Region	nt Start *	nt End *	% 16 hpi ^§^	% 48 hpi ^§^
Leader	530	585	0.16	0.18
Intron NS	586	2088	0.02	0.01
Exon Long	2089	2208	0.20	0.20
Exon Short	2209	2362	0.28	0.28
pAp1	2363	2841	0.14	0.10
pAp2	2842	3141	0.04	0.05
Exon VP1	2842	3223	0.02	0.03
Exon VP2	3224	4882	0.04	0.04
pAd	4883	5189	0.08	0.08
Terminal	5190	5213	0.02	0.03

* nt start and nt end indicate the genomic regions selected for HTS count; ^§^, percentage of reads mapped to the different genomic regions, normalized to the respective length.

**Table 4:** Frequency of alternative mRNA processing events at the indicated sites.

Processing	nt Start *	nt End *	% 16 hpi ^§^	% 48 hpi ^§^
Splicing				
D1-no splicing	585	586	0.08	0.03
D1-A1.1	586	2088	0.65	0.77
D1-A1.2	586	2208	0.27	0.20
D2-no splicing	2362	2363	0.69	0.61
D2-A2.1	2362	3141	0.05	0.09
D2-A2.2	2362	4882	0.26	0.30
Cleavage				
pAp1	2841	2842	0.38	0.48
pAp2	3141	3142	0.07	0.06
pAd	5190	5191	0.60	0.28

* nt start and nt end indicate the genomic positions selected for HTS count; ^§^, percentage of reads mapped to the different genomic regions, normalized to the respective alternative processing patterns.

## Data Availability

The original raw FASTQ reads presented in this study have been submitted to the European Nucleotide Archive, Accession PRJEB105312.

## Supplementary Materials

The following supporting information can be downloaded at: https://www.mdpi.com/article/10.3390/v18010039/s1, Supplemental File S1: B19V replication in sorted EPC; Supplemental File S2: DGS analysis in EPCs; Supplemental File S3: GSEA in EPCs, synchronic; Supplemental File S4: GSEA in EPCs, diachronic; Supplemental File S5: STRING analysis in EPCs.

## Abbreviations

The following abbreviations are used in this manuscript:

B19V	Parvovirus B19
PBMC	Peripheral Blood Mononuclear Cells
EPCs	Erythroid Progenitor Cells
HTS	High Throughput Sequencing

## Appendix A

**Table A1:** Distribution of markers other than erythroid in EPCs on day 8 of in vitro differentiation.

Population *		CD14	CD45	CD3	CD4	CD8	CD19
P1	**%**	1.46	10.45	2.63	0.05	0.86	5.73
P2	**%**	1.70	92.89	84.18	51.17	30.37	3.37

* P1 and P2 subpopulation of EPCs as defined in Figure 2. Direct labeling of cells by using the following antibodies: CD14PE/CD45FITC, BD Biosciences, Cat. N. 342408; CD3FITC, Beckman Coulter, Cat. N. A07746; CD4FITC/CD8PE, BD Biosciences, Cat. N. 349508; CD19FITC, Miltenyi, Cat. N. 130-113-168.

**Table A2:** Sequence strings used for selection and mapping of mRNAseq reads: strings containing the specific sequence as derived from processing of pre-mRNA at the indicated sites.

Region	nt Start *	nt End *	Sequence
Splicing			
D1-no splicing	585	586	GTGAGCTAACTAACAGGTATTTATACTACTTG
D1-A1.1	586	2088	GTGAGCTAACTAACAGATGCCCTCCACCCAGA
D1-A1.2	586	2208	GTGAGCTAACTAACAGGCGCCTGGAACACTGA
D2-no splicing	2362	2363	ACCAGTTTCGTGAACTGTTAGTTGGGGTTGAT
D2-A2.1	2362	3141	ACCAGTTTCGTGAACTGTGCAGCTGCCCCTGT
D2-A2.2	2362	4882	ACCAGTTTCGTGAACTCTACAGATGCAAAACA
Cleavage			
pAp1	2841	2842	TTGCTCGTATTAAAAATAACCTTAAAAACTCT TAACCTTAAAAACTCTCCAGACTTATATAGTC CCAGACTTATATAGTCATCATTTTCAAAGTCA
pAp2	3141	3142	TGGGAATAAATCCATATACTCATTGGACTGTA TACTCATTGGACTGTAGCAGATGAAGAGCTTT GCAGATGAAGAGCTTTTAAAAAATATAAAAAA
pAd	5190	5191	AAAATTTAGAAAAATAAACATTTGTTGTGGTT AACATTTGTTGTGGTTAAAAAATTATGTTGTT AAAAAATTATGTTGTTGCGCTTTAAAAATTTA

* nt start and nt end indicate the genomic positions selected for HTS count.

**Table A3:** Interaction network analysis via STRING.

Cluster Number		Gene Count	Clustering Coefficient	Protein Names	Reactome Pathways	Avg Log FC
2 hpi	1	35	0.68	SLC2A3, IGF2, MYC, HK2, PFKFB3, CCND1, CDKN2C, KLF4, SOCS3, ICAM1, CDKN1B, CXCR4, DDIT4, CISH, MMP9, SFN, CCNE1, ANGPTL4, CCN1, AREG, LIF, TOR1AIP2, SLC2A1, PIM1, CDKN1C, TXNIP, HES1, NAGK, ETV5, RASGRP1, SUV39H1, RPS6KA5, KDM3A, CITED2, TIPARP	Signal transduction Gene transcription Cytokine signaling Cell cycle	−0.83
	2	24	0.68	BHLHE40, BCL3, PTGS2, FOSL2, MAFB, JUN, FOS, NFKBIA, SMAD7, IER3, ATF3, HSPA5, NFIL3, TGFB3, PMAIP1, RUNX1, PLA2G4A, TRIB1, ID2, PRDM1, KLF10, MAFF, DDIT3, ERRFI1	Signal transduction PERK regulated gene expression	−1.73
	3	10	0.86	AURKA, RAD54L, SPC25, WEE1, FBXO5, FEN1, CCNF, POLH, DCLRE1B, POLL	Cell cycle, mitotic DNA Repair	0.75
	4	7	0.79	PLAUR, TLR8, NOD1, CCL2, CFB, PGF, FPR1	Innate immune system	−1.73
	5	6	0.90	DHX58, OAS2, RSAD2, TDRD7, ISG20, IRF9	Interferon alpha/beta signaling	0.52
	6	4	0.83	BARD1, BRCA2, CBX8, PSMC3IP	Cell cycle	−0.01
	7	3	1.00	UMPS, CMPK2, UPP1	Pyrimidine metabolism	0.45
	8	3	1.00	MT2A, KRT13, MT1E	Metal homeostasis	−3.77
	9	3	0.67	IRS2, LDLR, INSIG1	Lipid homeostasis	−1.96
	10	3	0.67	TNFAIP3, PELI1, ITGBL1	Regulation of TLR3/4 pathways	−0.10
16 hpi	1	15	0.82	CCL2, TLR2, CA12, SERPINE1, LOX, TIMP1, MMP9, PF4, TGFBI, ELANE, PLAU, PLAUR, MMP14, FOSL2, SELENBP1	Extracellular matrix organization Hemostasis	1.43
	2	6	0.85	PLEK, BCL2A1, MARCKS, IGSF6, FGR, PHLDA1	No match	1.78
	3	5	0.87	GPX3, HMOX1, MT2A, ALAD, MT1E	Cellular response to metal ion Oxidative stress response	−0.41
	4	4	1.00	CD74, IFI30, CTSS, HLA-DRA	MHC-II antigen presentation	1.56
	5	4	0.75	ACP5, CYP27A1, CH25H, MGLL	Synthesis of bile acids/salts	0.68
	6	4	0.83	SDC4, TGM2, GPC1, SDC2	Exostosis	0.75
	7	3	0.67	CCR1, CCL13, GRK5	Chemokine signaling pathway	1.95
48 hpi	1	87	0.65	PECAM1, VCAN, CD48, CCR1, SLAMF7, CCL2, IL1B, TNF, CTSK, C3AR1, SDC4, SDC1, TLR2, CD38, CD86, CCL5, CTSS, CXCR4, NR4A2, NR4A1, MMP9, PTGS2, SP1, ANPEP, S100A4, BIRC3, SLA, EPCAM, P2RX7, PTGER4, CMKLR1, IL32, TPH1, MMP14, IRF5, GPR183, CSF3R, KLRK1, PDCD1, PLAUR, C5AR1, LY96, TLR8, MSR1, PLAU, SELL, ITGA5, TNFRSF1B, TLR1, FPR1, IL1R2, ACE, OLR1, PPBP, IL1R1, IDO1, CASP1, SELP, FGF2, CXCL1, PF4, CD70, SLAMF1, IL3RA, NNMT, COL2A1, TGFBI, TNFSF9, IRAK2, CD55, JAG1, ADAMDEC1, CD14, BCL2L11, KCNN4, MARCO, ECM1, GNA15, PDPN, IL13RA1, TNFAIP2, IL6ST, ADAM9, GP1BA, ETV5, BANK1, CSF2RA	Immune system Signal transduction Cytokine signaling Innate immune system Signaling by interleukins Hemostasis	2.00
	2	16	0.78	CD74, NLRC5, PSMB9, SEC24D, CTSV, CTSF, IFI30, CTSH, HLA-B, CIITA, HLA-F, DOCK4, LGMN, HLA-DMA, HLA-DQA1, HLA-DRB1	Adaptive immune system MHC-II antigen presentation Cytokine signaling	1.85
	3	7	0.87	IFITM1, DDX60, XAF1, SAMHD1, EPSTI1, GBP4, ISG20	Interferon signaling Interferon alpha/beta signaling	0.75
	4	7	0.77	TARS1, ASNS, NUPR1, DDIT4, TRIB3, MTHFD2, CEBPG	Response of EIF2AK1 to heme/amino acid deficiency	−0.20
	5	5	0.87	SCD, FADS1, ACSL1, ACSL3, FABP3	Metabolism of lipids	0.90
	6	5	0.80	TFRC, TG, NCOA4, SLC7A11, TPD52	Ferroptosis	1.86
	7	4	0.83	ACTA2, SORBS3, FBLN5, MFAP5	No match	1.73
	8	4	1.00	APOA1, TF, SERPINA1, APOD	Platelet degranulation	3.63
	9	4	0.75	IGFBP4, STC2, HTRA1, PRG2	Insulin-like growth factor	1.48
	10	4	0.83	GPX3, GPX7, ALOX15B, GLRX	Detoxification of ROS	2.55
	11	4	0.75	LCK, LAT2, SH2B2, MAP4K1	Regulation of KIT signaling	2.20
2–48 hpi	1	98	0.70	PECAM1, VCAN, CD48, CCR1, CD83, ICOS, CCR7, SLAMF7, CXCL8, ICAM1, CCL2, FCER1G, IL1B, TNF, C3AR1, CXCL16, CD40, SDC4, ITGAM, SDC1, TLR2, CD38, CD86, CCL5, ITGB2, CTSS, CXCR4, TRAF1, SOCS3, TNFAIP3, NFKBIA, MMP9, NOD1, TIMP2, OSM, IL7, CISH, HBEGF, IL13, STAT4, CXCL1, IL15, CXCL2, IL3RA, IL13RA1, CSF3R, LIF, IL6ST, LGALS1, ANPEP, ALCAM, PLAU, TNFAIP2, IRF5, EDN1, APAF1, LTB, CXCL3, PLAUR, TLR8, IL1R2, LY96, IL18, CASP1, BIRC3, IRAK1, EPCAM, TRAF4, S100A9, CMKLR1, TPH1, KLRK1, C5AR1, MSR1, FPR1, OLR1, PPBP, IDO1, SELP, CCRL2, PF4, ITGA6, CD70, PRDM1, CR2, TXNIP, TGFBI, CD14, ECM1, ID2, PDPN, EMP1, MARCO, GP1BA, GSDME, MGAT1, BANK1, SERPINB6	Immune system Cytokine signaling in immune system Signal transduction Innate immune system	2.84
	2	20	0.82	CD74, CST7, KCND1, CTSV, CTSF, KCNA3, IFI30, CTSD, CTSH, SELENOP, HLA-B, CIITA, HLA-F, SEC61A1, DOCK4, LGMN, CTSL, HLA-DMA, HLA-DQA1, HLA-DRB1	Immune system Adaptive immune system MHC-II antigen presentation	1.79
	3	19	0.82	GADD45B, NR4A2, DUSP2, DUSP5, BTG2, NR4A1, FOSB, FOS, DDIT3, JUN, ATF3, MAFB, BCL2L11, TRIB1, CDK5R1, ERRFI1, MAFF, ATF1, ETV5	Signal transduction Generic transcription pathway	2.10
	4	16	0.90	IFITM1, IRF7, USP18, TDRD7, SAMHD1, ISG20, IRF9, XAF1, EPSTI1, CMPK2, DHX58, RTP4, MX2, IFI44, RSAD2, SP110	Immune system Interferon alpha/beta signaling	0.94
	5	11	0.85	COL5A2, SERPINH1, PLOD3, COL6A3, COL2A1, COL7A1, COL6A2, AEBP1, EFEMP2, FBLN5, MFAP5	Extracellular matrix organization	1.34
	6	10	0.91	HSPA1A, HSPA9, HSPD1, DNAJB1, HSPA13, HSPA4L, HSPA2, STIP1, TIMM10, NUP58	Cellular responses to stress	−0.34
	7	10	0.84	MYC, FOXO4, FOXO3, NEDD9, SFN, CCNE1, NOTCH1, CEBPA, PIM1, RNF4	Generic transcription pathway	0.49
	8	9	0.80	FBXO5, AURKA, PLK3, CDC27, CALU, PRC1, PHLDA1, CPEB2, INCENP	Cell cycle, mitotic	−0.79
	9	9	0.81	GNG2, GNAI3, PTGER4, PREX1, APLNR, PTGIR, GABBR1, GNA15, ADRA2B	GPCR downstream signaling	1.65
	10	8	0.85	CALM3, MARCKS, BASP1, CCP110, ATP2B1, ATP2A1, GEM, AKAP12	Signaling by Rho GTPases Reduction of cytosolic Ca++ s	0.32
	11	7	0.78	PLEK, SPHK1, BRPF3, DGKA, DGKH, ARHGEF3, DAPP1	Platelet activation, signaling, and aggregation	0.74
	12	7	0.79	APOA1, PLA2G7, APOD, ABCA2, PCSK9, TF, ADAMDEC1	Transport of small molecules	4.46
	13	7	0.88	WIPF1, WASL, CDC42EP4, CDC42EP2, ARHGAP4, PAK4, CYFIP2	RHO GTPase cycle	−0.53
	14	6	0.75	MAPRE3, MAPRE1, APC, CLIP2, MAP7, NUPR1	No match	0.54
	15	6	0.86	TFB2M, DNTTIP2, BYSL, NIP7, GRWD1, YRDC	No match	−1.16
	16	6	0.76	CFB, CFD, C3, C1S, F13B, MASP2	Initial triggering of complement	3.34
