# Supplementary material for: Parvovirus B19 and Cellular Transcriptome Dynamics in Differentiating Erythroid Progenitor Cells
Source: Viruses. 2025 Dec 25;18(1):39. doi: 10.3390/v18010039 (PMC12846583; doi:10.3390/v18010039)
Supplement: Supplementary file 1 [file viruses-18-00039-s001.zip › B19V Viruses supplementary File 1.pdf]

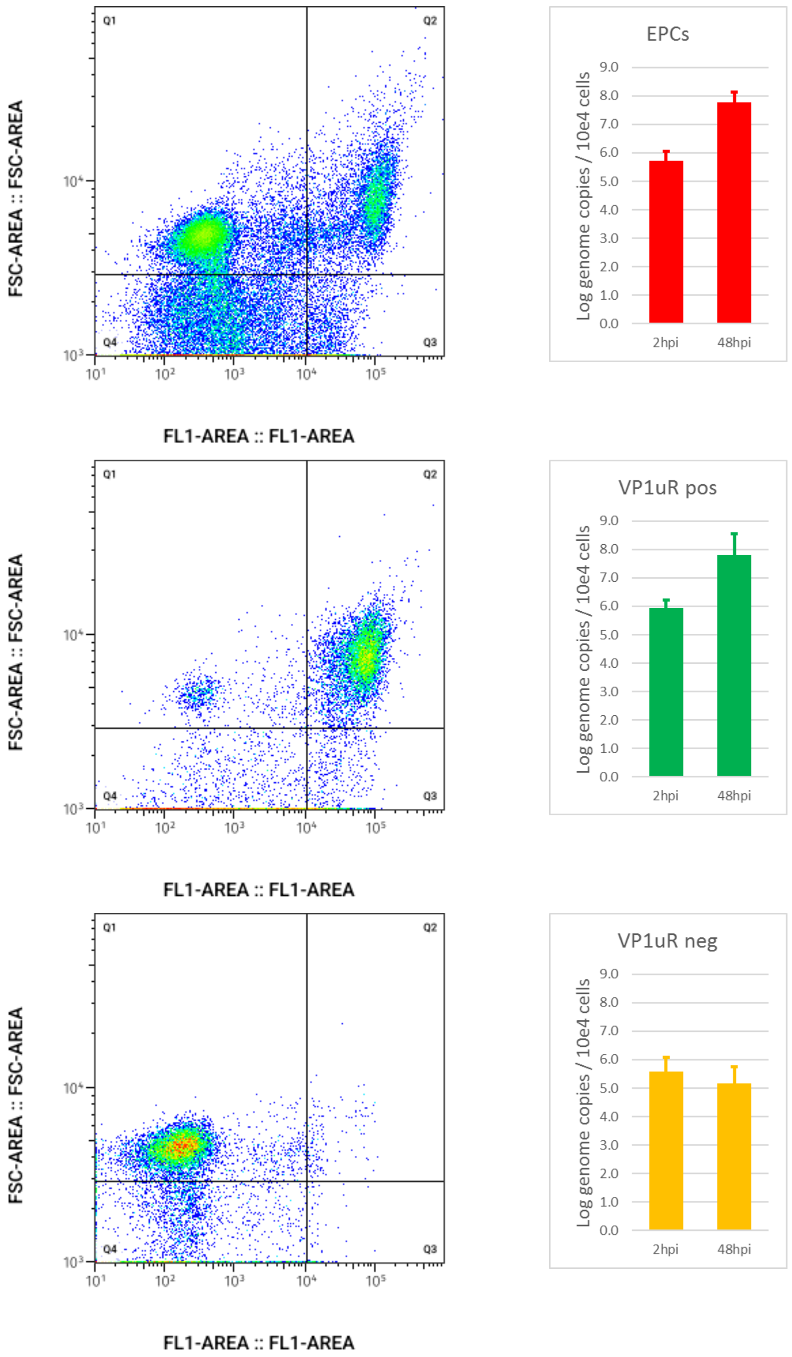

FACS and qPCR analysis of different population subsets in EPC.

EPCs at day 8 of in vitro growth and differentiation were analyzed based on the VP1uR expression; cells were then sorted as either VP1uR positive or negative, by a S3e™ Cell Sorter (Bio-Rad Laboratories, Inc., California, USA). Dot-plot graphs of flow cytometric detection of VP1uR in unsorted (top), positive sorted (middle) and negative sorted (bottom) cells (FlowJo, Ashland, OR, USA). Aside, quantification of viral DNA in unsorted (top), positive sorted (middle) and negative sorted (bottom) cells infected with B19V and collected at 2 and 48 hpi. Data are shown as means +SD from independent experiments performed in triplicate.
